# Mitigating Effect of Estrogen in Alzheimer’s Disease-Mimicking Cerebral Organoid

**DOI:** 10.3389/fnins.2022.816174

**Published:** 2022-03-24

**Authors:** Jennifer Yejean Kim, Hyunkyung Mo, Juryun Kim, Jang Woon Kim, Yoojun Nam, Yeri Alice Rim, Ji Hyeon Ju

**Affiliations:** ^1^Department of Biology, Georgetown University, Washington, DC, United States; ^2^CiSTEM Laboratory, Catholic iPSC Research Center, College of Medicine, The Catholic University of Korea, Seoul, South Korea; ^3^YiPSCELL, Inc., Seoul, South Korea; ^4^Division of Rheumatology, Department of Internal Medicine, Seoul St. Mary’s Hospital, Institute of Medical Science, College of Medicine, The Catholic University of Korea, Seoul, South Korea

**Keywords:** Alzheimer’s disease, induced pluripotent stem cells, cerebral organoid, estrogen, amyloid-beta

## Abstract

Alzheimer’s disease (AD) is the most common condition in patients with dementia and affects a large population worldwide. The incidence of AD is expected to increase in future owing to the rapid expansion of the aged population globally. Researchers have shown that women are twice more likely to be affected by AD than men. This phenomenon has been attributed to the postmenopausal state, during which the level of estrogen declines significantly. Estrogen is known to alleviate neurotoxicity in the brain and protect neurons. While the effects of estrogen have been investigated in AD models, to our knowledge, they have not been investigated in a stem cell-based three-dimensional *in vitro* system. Here, we designed a new model for AD using induced pluripotent stem cells (iPSCs) in a three-dimensional, *in vitro* culture system. We used 5xFAD mice to confirm the potential of estrogen in alleviating the effects of AD pathogenesis. Next, we confirmed a similar trend in an AD model developed using iPSC-derived cerebral organoids, in which the key characteristics of AD were recapitulated. The findings emphasized the potential of estrogen as a treatment agent for AD and also showed the suitability of AD-recapitulating cerebral organoids as a reliable platform for disease modeling and drug screening.

## Introduction

Alzheimer’s disease (AD) is the most common neurodegenerative disorder characterized by the gradual loss of cognitive function, leading to progressive disruptions in basic functions such as walking, swallowing, memory, and attention (Goedert and Spillantini, 2006; Montine et al., 2012; Dubois et al., 2016) primarily characterized by extracellular accumulation of amyloid-beta (Aβ) peptides and formation of neurofibrillary tangles from the intraneuronal accumulation of hyperphosphorylated proteins (Bancher et al., 1989; Montine et al., 2012; Penney et al., 2020). The aggregation of Aβ proteins increases under oxidative stress (Cho et al., 2016), thereby promoting proinflammatory responses (Yang et al., 2015), inducing non-functional synaptic plasticity, deregulating intracellular signaling pathways, and initiating neuronal apoptosis (Roberts et al., 1994; Zheng et al., 2017).

According to the Alzheimer’s Association, two of three people diagnosed with AD are women (Alzheimer’s Association, 2017^1^). Such could be simply attributable to women’s longer expected life span when compared to men (Alzheimer’s Association, 2016^1^) as well as decreased level of estrogen in postmenopausal women. A decrease in the level of estrogen, neuroactive steroid hormones associated with memory, cognition, and sexual conduct, in the postmenopausal state is associated with a heightened disease risk (Hojo et al., 2008; Wharton et al., 2011). They are some of the most important contributors to cognitive function through hippocampal neurogenesis (Sager et al., 2018). The level of estradiol, the most common form of estrogen, declines in the postmenopausal state, and eventually becomes lower than that in men (Khosla et al., 2001). Estrogen deficiency in postmenopausal women has been linked to the pathogenesis of AD (Barnes et al., 2005).

Estrogen exerts neuroprotective effects by decreasing Aβ and glutamate toxicities (Xu et al., 1998) and suppressing tau protein hyperphosphorylation (Goodenough et al., 2005; Zhang et al., 2008; Lee et al., 2014). Estrogen also improves synaptic plasticity, helps maintain the neurotrophic components, and reduces inflammation in the brain (Correia et al., 2010; Pompili et al., 2012). While certain studies could not confirm the abovementioned positive effects of estrogen (Henderson, 2014), other studies provided experimental evidence that women who undergo hormone replacement therapy are at a lower risk of AD (Tang et al., 1996; Daniel and Bohacek, 2010).

Many studies have conducted *in vitro* and *in vivo* experiments on AD and provided methods for curing the disease; however, the effectiveness has not been confirmed in clinical trials (Zahs and Ashe, 2010). The modeling platforms used have certain limitations, the most important one being that the transgenic mice commonly used to study AD do not completely recapitulate AD pathogenesis. Hence, alternative disease modeling platforms, such as those developed using induced pluripotent stem cell (iPSC)-derived organoids, are necessary to study the pathological mechanisms of AD that involve central lesions. While there are primarily three methods used for modeling AD using cerebral organoids (Koch et al., 2012; Vazin et al., 2014; Huang et al., 2017; Pavoni et al., 2018), these models exhibit low reproducibility and homogeneity, and thereby fail to accurately mimic AD pathogenesis (Papaspyropoulos et al., 2020).

Since their introduction in 2006, iPSCs have been used in different fields of science and medicine. Cerebral organoids, which recapitulate different parts of the brain, have been widely used to study different neurodevelopmental and neurodegenerative disorders since their introduction in 2013.

In this study, we first confirmed the effect of estrogen in an ovariectomized mouse model of AD. Next, we generated healthy iPSC-derived cerebral organoids (iCOs) and treated them with Aβ peptides to effectively induce a controlled AD-like environment. The relationship between estrogen and iPSC-derived neurons has been investigated using a two-dimensional platform (Shum et al., 2015). However, to the best of our knowledge, experiments investigating the effects of estrogen on neurons have not been performed using a three-dimensional platform. Additionally, to evaluate the effect of estrogen on AD-like iCOs, we administered relatively low and high doses of estradiol and investigated its dose-dependent effects. Our findings showed the potential of Aβ-treated iCOs as a reliable disease modeling and drug screening platform for studying AD and suggested the suitability of estrogen as a therapeutic candidate for AD.

## Materials and Methods

### Ethics statement

All procedures involving experimental animals were performed in accordance with the Laboratory Animals Welfare Act, the Guide for the Care and Use of Laboratory Animals, and the Guidelines and Policies for Rodent Experiments provided by the Institutional Animal Care and Use Committee of the School of Medicine, The Catholic University of Korea. The study was approved by the Institutional Review Board of The Catholic University of Korea (CUMC 2020-0296-04).

### Animals and Treatment

Female 5xFAD mice (10 mice, weight 20–30 g, 4 to 5 weeks of age) and female C57BL/6 mice (5 mice, weight 20–30 g, 5 weeks of age) were maintained in a pathogen-free, strictly controlled environment. In this study, three groups of mice were studied: one control group consisted of five female C57BL/6 mice, one disease control group consisted of five female 5xFAD mice, and one experimental group consisted of five female 5xFAD mice. The body weight of each mouse was monitored weekly. The mice were housed individually, had free access to food and water, and were maintained under the abovementioned conditions until 9 weeks of age, after which ovariectomy was performed. All surgical manipulations were conducted under anesthetization (isoflurane, 2–5% *via* inhalation). The mice were allowed to recover for 2 weeks and injected with gentamicin (5 mg/kg, subcutaneous injection) and ketoprofen (5 mg/kg, subcutaneous injection) daily for 1 week to prevent infection and alleviate pain. When aged 11 weeks, the mice were injected with estrogen (Merck, Darmstadt, Germany, Cat# E8875) 10 μg/kg/day, 5 days/week, 6 weeks). When aged 17 weeks, the mice were made to perform the Barnes Maze test; thereafter, the mice were sacrificed under 1.5∼4.5% isoflurane, and their tissues were used for histological analyses.

### Barnes Maze Test

The Barnes maze test was conducted to assess the spatial learning and memory of the AD mice. The paradigm comprised a large, 92 cm-wide circular board with 18 holes, with one of them as the “target.” The “target” contained the food (sweet flavored chips) from the cage in which the mice were housed.

During the two pre-training trials, the mice were placed in the middle of the maze inside a transparent beaker for 1 min. This allowed the mice to familiarize themselves with their surroundings, including three signs on the wall. After 1 min, the mice were guided to the target and allowed to remain in the target for 2 min. The pre-trial training was conducted two times over 2 days before the trial.

At the beginning of the trial, the mice were placed in the middle of the maze and allowed to locate the target for 3 min. The process was recorded using SMART3.0 (Panlab, Harvard Apparatus). After the final trial was complete, the data collected from each trial were summarized. We have collected three types of data: the time it takes for the mice to make their first entry to the target (first entry to target), the distance the mice walked within the target (distance in target), and the time spent outside of the target (time in error).

### Induced Pluripotent Stem Cells Culture

Peripheral blood mononuclear cell-derived hiPSCs were generated and characterized in our previous studies (Kim et al., 2016). The blood was collected from PBMC-derived hiPSCs (female, age 30-35, passage 18-25) were seeded on vitronectin (Gibco, Carlsbad, CA, United States, Cat #A14700) coated dishes in a 10% CO_2_ environment at 37°C. The cells were cultured in Essential 8 medium (Gibco Cat #A1517001) that was replaced each day until differentiation was induced.

### Induced Pluripotent Stem Cells-Derived Cerebral Organoid Differentiation

To induce the differentiation of iPSCs into iCOs, we adapted a protocol reported by Lancaster and Knoblich (2014). The iPSCs were initially suspended in low-bFGF hESC medium composed of 40 mL of DMEM-F12 (Gibco Cat #11320023), 10 mL of knock-out serum (Gibco Cat #10828010), 1.5 mL of fetal bovine serum (Gibco Cat #10437028), 0.5 mL of GlutaMax (Gibco Cat #35050061), 0.5 mL of MEM-NEAA (Gibco Cat #11140050), and 3.5 μL of 2-mercaptoethanol (Sigma Aldrich, St. Louis, MO, United States). bFGF (R&D systems, Minneapolis, MN, United States) was added at a final concentration of 4 ng/mL, and ROCK inhibitor (Peprotech, Rocky Hill, NJ, United States) was added at a final concentration of 50 μM. The formation of embryonic bodies (EBs) was induced in the suspended iPSCs by culturing in an untreated U-shaped 96-well plate. Approximately 9 × 10^3^ cells were dispensed in each well and centrifuged. The day of seeding was considered as day 0. The medium from each well was aspirated gently on days 2 and 4 and replaced with 150 μL of fresh low-bFGF hESC medium. From day 0, the EBs were incubated at 37°C in 5% CO_2_. On day 6, each EB was transferred to an untreated 24-well plate containing 500 μL of neural induction medium consisted of DMEM-F12 with 1% N2 supplement (Gibco Cat #17502001), 1% GlutaMax, and 1% MEM-NEAA. Five hundred microliters of media were added on day 8. On day 11, the EBs were embedded in Matrigel (Corning, Corning, NY, United States, Cat #356234). Matrigel with the embedded EBs was hardened at 37°C in 5% CO_2_ for 2–30 min. The EBs embedded in Matrigel were cultured in cerebral organoid differentiation media without vitamin A until day 15. From day 15, the EBs were cultured in cerebral organoid differentiation media containing vitamin A in untreated 6-well plates (VWR, Radnor, PA, United States, Cat #10062-892) on a rocker at 37°C in 5% CO_2_. The organoids were cultured in fresh differentiation media every 3 days until they were harvested on day 60. From day 61, organoids were treated with estrogen (Merck, Darmstadt, Germany, Cat# E8875) every three days in different concentrations (1nM and 10nM) for 30 days.

### Real-Time RT-PCR

Samples were collected and stored at −80°C until use. mRNA was extracted from the samples using TRIzol reagent (Life Technologies, Carlsbad, CA, United States). Two micrograms of extracted mRNA were used to synthesize cDNA using the RevertAid First Strand cDNA Synthesis Kit (Thermo Fisher Scientific, Carlsbad, CA, United States, Cat #K1622). The PowerSYBR Green PCR Master Mix (Applied Biosystems, Waltham, MA, United States, Cat #436759) was used to measure gene expression using the StepOnePlus Real-Time PCR System (Applied Biosystems). Total of 40 cycles were used per trial: 15 seconds in 95°C, then 30 s in 60°C, followed by 45 s in 72°C. The relative mRNA levels were normalized to those of *GAPDH*. The primers used are listed in Table 1.

**TABLE 1:** List of primers used for quantitative real-time polymerase chain reaction (human).

Gene	Organism	Forward Primer	Reverse Primer
*GAPDH*	Human	CTGTTGCTGTAGCCAAATTCGT	ACCCACTCCTCCACCTTTGA
*OCT4*	Human	ACCCCTGGTGCCGTGAA	GGCTGAATACCTTCCCAAATA
*FOXG1*	Human	AGGAGGGCGAGAAGAAGAAC	TCACGAAGCACTTGTTGAGG
*TBR1*	Human	GGGCTCACTGGATGCGCCAAG	TCCGTGCCGTCCTCGTTCACT
*PROX1*	Human	GCAGTAGTTTCCTCCTGACCG	TCTCTGTGTTGGTGCCGCC
*FZD9*	Human	AAAGTCAAATGTACTCCGCAAGC	CTGGGAAATTATGGTTGCTCCT
*MAP2*	Human	GGAGACAGAGATGAGAATTCC	GAATTGGCTCTGACCTGGT
*NeuN*	Human	GCGGCTACACGTCTCCAACAT	ATCGTCCCATTCAGCTTCTCCC
*TUJ1*	Human	GGCCTTTGGACATCTCTTCA	ATACTCCTCACGACCTTGC
*vGlut1*	Human	CCATGACTAAGCACA	AGATGACACCTCCATAGTGC
*Nestin*	Human	ACCAAGAGACATTCAGACTCC	CCTCATCCTCATTTTCCACTCC
*APP*	Human	AACCCTACGAAGAAGCCACA	TTCTCATCCCCAGGTGTCTC
*ADAM10*	Human	AATTCTGCTCCTCTCCTGGGC	TATGTCCAGTGTAAATATGAGAGG

### Sample Preparation for Cerebral Organoids and Mouse Brains

The harvested samples were stored overnight in 4% paraformaldehyde (Tech & Innovation, Chuncheon, Republic of Korea, Cat #BPP-9004) at 4°C and dehydrated by overnight immersion in 15% sucrose and then in 30% sucrose at 4°C. After dehydration, the samples were immersed overnight in OCT compound (Sakura Finetek United States, Torrance, CA, United States, Cat #4583) and gradually frozen. The frozen samples were stored at −80°C until use. The samples were sectioned using a Cryo Microtome (Leica Biosystems, Wetzlar, Germany), and the slides were stored at −20°C until staining.

### Hematoxylin and Eosin (H&E) Staining

The sample slides were dried overnight at room temperature (22°C) before fixation in cold acetone for 10 min. The slides were incubated in filtered Harris’ hematoxylin (Sigma Aldrich, Cat #HHS32-1L) for 15 min, dipped in 1% HCl-EtOH, neutralized with 0.2% ammonia water, and then dipped in eosin three times for counterstaining.

### Immunohistochemical Staining

The sample slides were dried overnight at room temperature before fixation in cold acetone for 10 min. Endogenous peroxidase activity was blocked by treating with 0.3% hydrogen peroxide. Since the antibodies used were isolated from a mouse host, the Mouse on Mouse (M.O.M) Immunodetection Kit (Vector Laboratories, Burlingame, CA, United States, Cat #BMK-2202) was used. The slides were initially blocked with the M.O.M Mouse IgG Blocking Reagent diluted in 1% of bovine serum albumin (BSA) in 1% phosphate buffered saline (1% PBA) for 1 h, which was followed by protein blocking with the M.O.M. Protein Concentrate diluted in 1% phosphate-buffered saline (PBS) for 5 min. The primary antibodies were diluted in the protein concentrate dilutant used for protein blocking, and the slides were treated overnight with the primary antibodies at 4°C. The next day, the slides were incubated for 10 min in a 0.5% biotinylated secondary antibody solution at room temperature. After washing with 1% phosphate-buffered saline/tween (PBST), the slides were treated with ABC Reagent (Vector Laboratories, Cat #PK-7100) for 10 min, and then with 3,3′-diaminobenzidine solution (DAB) (Vector Laboratories, Cat #SK-4100) for 1 min. The slides were washed and counterstained with Mayer’s hematoxylin for 1 min, dehydrated, and cleared. The slides were then mounted and visualized using a bright-field microscope. β-amyloid quantification was performed using ImageJ program. The images collected from bright-field microscope were converted into 8-bit images and then threshold was adjusted to select the stained areas of β-amyloid. Once the threshold is applied, the mean area was measured under “analyze” tab.

### Immunofluorescence Assay

The sample slides were washed with 1 × tris-buffered saline/tween (TBST) and quenched using 1 × citrate buffer. Subsequently, the sectioned samples were permeabilized using TBS 0.1% Triton X-100 and blocked with 0.1% Triton X-100 (Biosesang, Seongnam, Republic of Korea Cat #TR1020-500-00) mixed with 10% normal horse serum. The slides were treated overnight with primary antibodies at 4°C (refer to Table 2 for the list of antibodies used). Next, the samples were washed using 1 × TBST and then treated with Alexa Fluor 594-(1/400; Life Technologies, Carlsbad, CA, United States) and 488-(1/400; Life Technologies) conjugated secondary antibodies diluted in PBA and incubated for 1 h at RT avoiding light. 4′,6-diamidino-2-phenylindole (DAPI) was used for nuclear staining. After DAPI staining, samples were mounted and stored at 4°C until the immunofluorescence assay was conducted. The thickness of cerebral organoid samples was 10 um and the thickness of mouse brain samples was 20 um. Images were obtained using confocal microscopy. Pinhole size of 69 um was used when gathering data.

**TABLE 2:** List of primary antibodies used for the immunofluorescence assay.

Antibody	Brand	Catalog Number	Dilution
SOX2	Abcam (Cambridge, United Kingdom)	Abcam Cat# ab92484, RRID:AB_10585428	1:500
TUJ1	GeneTex (Irvine, CA, United States)	GeneTex Cat# GTX631836, RRID:AB_2814952	1:500
MAP2	Santa Cruz Biotechnology, Inc. (Dallas, TX, United States)	Santa Cruz Biotechnology Cat# sc-74421, RRID:AB_1126215	1:500
TBR1	Abcam	Abcam Cat# ab31940, AB_2200219	1:500
FOXG1	Abcam	Abcam Cat# ab18259, RRID:AB_732415	1:500
PROX1	Millipore (Burlington, MA, United States)	Millipore Cat# MAB5654, RRID:AB_2170714	1:500
B-amyloid	Santa Cruz Biotechnology, Inc.	Santa Cruz Biotechnology Cat# sc-374527, RRID:AB_10988723	1:100

### Western Blotting

Proteins were extracted from the samples using a protein extraction buffer (Thermo Scientific, Cat #78510) mixed with 100 × phenylmethylsulfonyl fluoride (PMSF) and a single cOmplete Mini, EDTA-free protease inhibitor cocktail tablet (Roche Diagnostics, Basel, Switzerland, Cat #22836170001). The homogenized samples were centrifuged, and the supernatants were collected. The protein concentration was measured using the Bradford assay (Bio-Rad, Cat #5000205). Equal quantities of proteins were loaded onto an SDS-polyacrylamide gel and electrophoretically transferred onto a nitrocellulose membrane (Bio-Rad Laboratories, Hercules, CA, United States, Cat #1704158). The membranes were treated overnight at 4°C with primary antibodies (refer to Table 3 for the list of antibodies used) and then for 1 h with a horse radish peroxidase-conjugated secondary antibody. The membranes were developed using Amersham Imager 600. The bands were measured using ImageJ program. The bands were selected by drawing a frame around them using the “rectangle” tool. The area was assigned and then profile plot of the lane was acquired using “plot lanes” tool. Each peak in the profile plot represents the relative intensity of the bands in the selected lane. Straight line selection tool was used in order to enclose each peak, then the wand tool was used to quantify the intensity.

**TABLE 3:** List of antibodies used for Western blotting.

Antibody	Brand	Catalog Number
APP	Sigma Aldrich (St. Louis, MO, United States)	Sigma-Aldrich Cat# A8717, RRID:AB_258409
pTAU	Cell Signaling Technology (Danvers, MA, United States)	Cell Signaling Technology Cat# 39357, RRID:AB_2799152
ADAM10	Santa Cruz Biotechnology, Inc.	Santa Cruz Biotechnology Cat# sc-48400, RRID:AB_626635
GAPDH	Santa Cruz Biotechnology, Inc.	Santa Cruz Biotechnology Cat# sc-32233, RRID:AB_627679

### Statistical Analysis

Statistical analyses were performed using GraphPad Prism version 9. Data are presented as mean and standard error of the mean (represented by the error bars). Student’s *t*-tests were used to compare the differences between the groups and to determine statistical significance. Two-tailed *P*-values were calculated using the *t*-test. Differences with *P* < 0.05, *P* < 0.01, and *P* < 0.001 were considered statistically significant.

## Results

### Estrogen Improved the Behavioral Performance of Ovariectomized Mice With Alzheimer’s Disease

**FIGURE 1:**
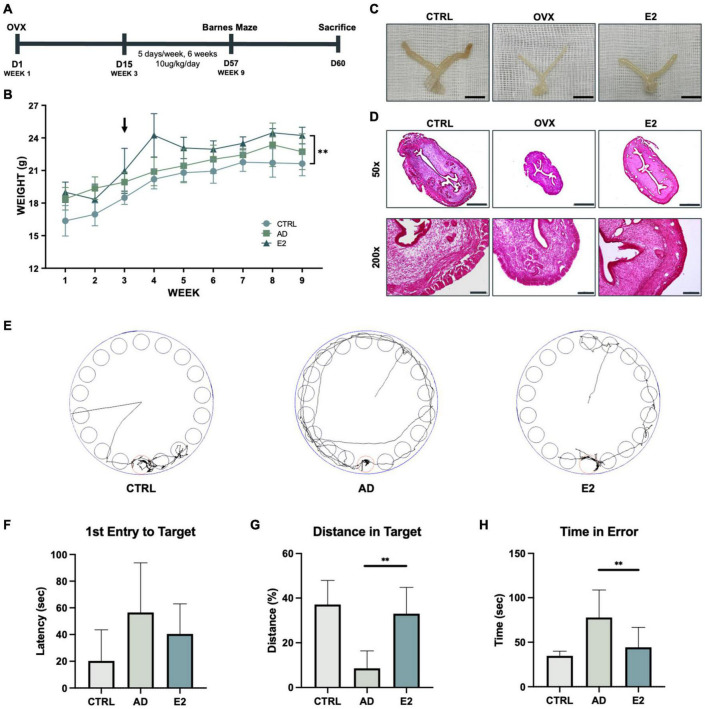
Estrogen treatment of an Alzheimer’s disease model developed using ovariectomized mice improves behavioral performance. **(A)** Schematic representation of the animal experiment, involving the induction of the postmenopausal state in the Alzheimer’s disease model of mice and treatment with estrogen. **(B)** Growth curves of control mice and ovariectomized (OVX) mice treated with estrogen. Arrows indicate the time of OVX operation and the starting point of estrogen injection. **(C)** Uteri of control (C57BL/6), OVX, and OVX + E2 mice. Scale bar, 1 cm. **(D)** Hematoxylin and eosin staining of mice uterine tissues. Scale bar, 500 μm for 50 × ; 100 μm for 200 ×. **(E)** Monitoring of mice from each group for 3 min (180 s). **(F)** Graph showing the average latency of first entry to target in seconds. **(G)** Graph showing the average distance traveled in the target relatively. **(H)** Graph showing the average time spent by the mice outside the target zone in seconds. *t*-tests were used for data analysis (^**^P < 0.01). Values are presented as mean ± standard error of the mean.

To confirm the effect of estrogen in AD, ovariectomy was performed on 5xFAD mice, a transgenic mouse model of AD (Figure 1A). To determine whether the ovariectomy was successful, the body weight of each mouse was measured weekly. The body weights of ovariectomized 5xFAD mice were higher than those of mice from the other groups, which indicated successful menopause induction (Sasayama et al., 2017; Figure 1B). The uteri of mice from each group were examined to confirm the success of estrogen delivery. The uterine thickness of ovariectomized control mice (OVX) was considerably lesser than that of OVX mice treated with estrogen (OVX + E2) (Figure 1C). Additionally, the uteri of OVX + E2 mice and healthy control mice were of similar thickness. H&E staining of coronal dissections of the uteri showed the thinned uterine walls of OVX mice and the thicker uterine walls of OVX + E2 mice (Figure 1D). These results suggested that ovariectomy was successfully performed in AD mice and menopause was effectively induced.

Following estrogen treatment for 6 weeks, the Barnes maze test was performed to examine the spatial learning and memory capacities of the mice (Rosenfeld and Ferguson, 2014). The mice were digitally monitored for 3 min (Figure 1E). 5xFAD mice treated with estrogen (OVX + E2) took less time to initiate their first entrance (latency of first entry to target), but the difference was not statistically significant (Figure 1F). Additionally, mice with AD spent significantly lesser time in the target compared to mice treated with E2, for longer distance the mice walked within the target signifies longer time the mice spent within the target (Figure 1G). The time spent in the error zone was also analyzed. As observed previously, mice with AD spent more time outside the target than mice treated with E2 (Figure 1H). Hence, the results showed that estrogen was effectively delivered in the mice and suggested the beneficial effects of estrogen on ovariectomized mice.

### Protein Expression of Alzheimer’s Disease -Specific Markers in Mice Brain Tissues Decreased in Estrogen-Treated Ovariectomized Mice

**FIGURE 2:**
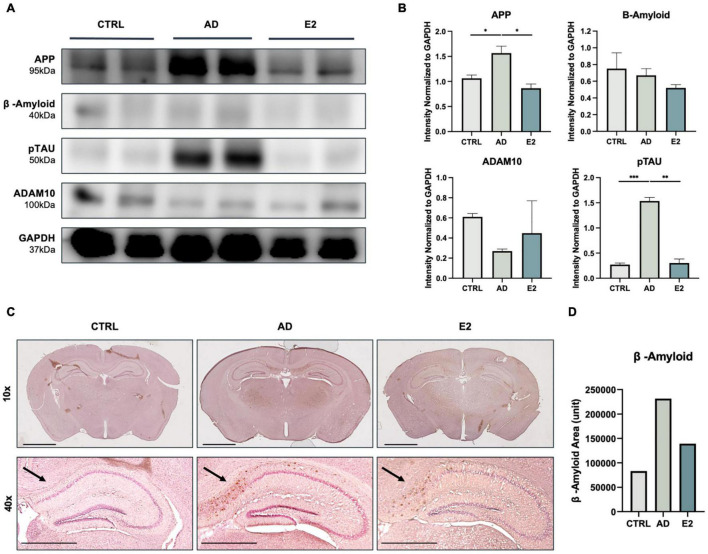
Histological analyses of brain tissues from control, 5xFAD, and ovariectomized 5xFAD model mice treated with estrogen. **(A)** Western blotting for the measurement of amyloid precursor protein (APP), amyloid-beta (Aβ) protein, phosphorylated tau (TAU) protein, and ADAM10 protein levels in each group. **(B)** Quantification in western blotting assay with protein levels normalized to that of GAPDH. **(C)** Immunohistochemical assay showed Aβ plaque accumulation in mouse brains, with significant clustering in the cortical and hippocampal regions. Scale bar, 2000 μm for 10x, 1000 μm for 40x. **(D)** Quantification of Aβ-positive areas, measured using ImageJ. *t*-tests were used for data analysis (*P < 0.05, ^**^P < 0.01, ^***^P < 0.001).

To confirm the effect of estrogen on mice with AD, we measured the expression of AD-specific markers in the brain tissues of sacrificed mice. The APP levels were significantly higher in the AD mice than in the control mice (t(2) = 4.675, *p* = 0.02). Conversely, the APP levels were significantly lower in the ovariectomized, estrogen-treated mice (E2 group) than in the AD mice (t(2) = 6.156, *p* = 0.01). Similarly, the E2-treated mice showed lower Aβ protein expression than the AD mice. Additionally, the phosphorylated tau protein (pTAU) levels in the E2 group was significantly lower than that in the AD group (t(2) = 16.35, *p* = 0.002), indicating the potential suppressive effect of estrogen on tau phosphorylation. We also measured the protein expression of *ADAM10*, which is the primary gene encoding alpha secretase. Soluble APPs, which do not form plaques and are thus non-neurotoxic, were found to be activated to a greater extent upon treatment with estrogen (Amtul et al., 2010). The *ADAM10* protein levels were higher in the E2 group than in the AD group (Figure 2A). The measured protein level from each analysis normalized to the GAPDH level is shown in Figure 2B. The presence and location of Aβ plaques were confirmed in the brain tissue samples. Compared to the control mice, the AD mice showed the apparent accumulation of Aβ plaques, and the accumulation was particularly high in the hippocampal region. Meanwhile, the accumulation was lower in the E2 mice than in the AD mice (Figure 2C). The measurement of Aβ-positive areas is shown in Figure 2D. The findings suggested the role of estrogen in suppressing Aβ peptide-induced neurotoxicity.

### Generation of Cerebral Organoids Using Induced Pluripotent Stem Cells

**FIGURE 3:**
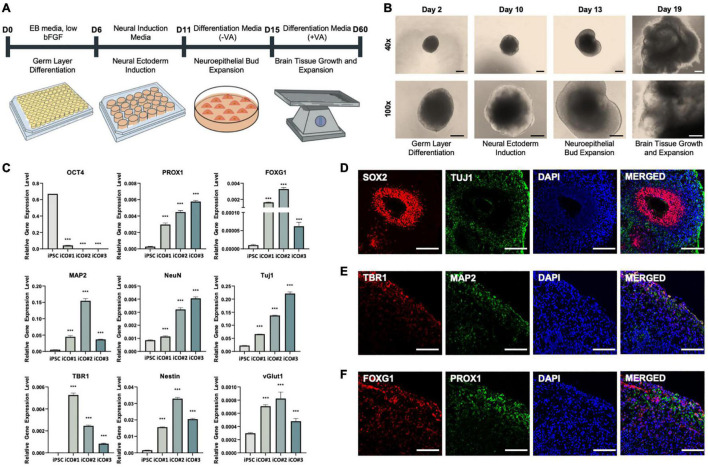
Successful differentiation of cerebral organoids from induced pluripotent stem cells obtained from healthy patient. **(A)** Differentiation of cerebral organoids derived from induced pluripotent stem cells. **(B)** Images of cerebral organoids in each step of the experiment. **(C)** Gene expression normalized to that of *GAPDH*: pluripotency marker *OCT4*, hippocampal marker *PROX1*, forebrain marker *FOXG1*, neuronal markers *MAP2*, *NeuN*, and *TUJ1*, deep-layer neuron marker *TBR1*, neural progenitor cell marker *Nestin*, and glutamate transporter marker *vGLUT1*. Immunofluorescence assay showing the co-localization of **(D)** early-stage neuronal marker SOX2 and neuronal cell-body marker TUJ1, **(E)** deep-layer neuron marker TBR1 and neuronal dendrite marker MAP2, and **(F)** forebrain marker FOXG1 and hippocampal marker PROX1, in the cerebral organoids. *t*-tests were performed for data analysis (***P < 0.001). Values are presented as mean ± standard error of mean. Scale bars, 200 μm.

**FIGURE 4:**
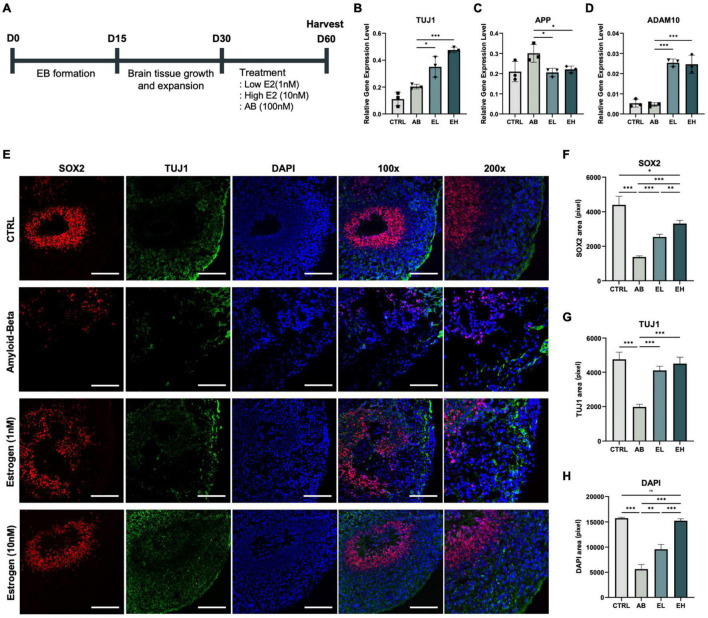
Estrogen treatment of cerebral organoids co-stimulated with amyloid-beta (Aβ) enhances the expression of neuronal markers. **(A)** Schematic representation of the cerebral organoid generation process and treatment with Aβ peptide and estrogen. Gene expression normalized to that of *GAPDH*: **(B)** neuronal markers *TUJ1*, **(C)** amyloid precursor protein marker *APP*, and **(D)** alpha-secretase marker *ADAM10*. **(E)** Immunofluorescence assay showing the co-localization of early-stage neuronal marker SOX2 and neuronal markers TUJ1 in samples obtained from experimental and control mice. **(F-H)** Areas that stained positive for DAPI, SOX2, and TUJ1 were measured using ImageJ. CTRL, control; Aβ, cerebral organoids treated only with 100 nM Aβ; EL, cerebral organoids treated with 100 nM Aβ and 1 nM estrogen; EH, cerebral organoids treated with 100 nM Aβ and 10 nM estrogen. *t*-tests were performed for data analysis (*P < 0.05, ^**^P < 0.01, ^***^P < 0.001). Values are presented as mean ± standard error of mean. Scale bars, 200 μm.

A schematic representation of the iCO differentiation process is provided in Figure 3A. The morphological characteristics of the iCOs were confirmed at each step of the protocol (Figure 3B). Robust cell expansion was confirmed on days 13 and 19 of differentiation. The gene expression levels were measured in the generated iCOs (Figure 3C). The expression of the pluripotency marker *OCT4* was significantly lower in iCOs than in iPSCs. The iCOs expressed various markers that are expressed in different regions of the brain, such as the hippocampal marker *PROX1* and the forebrain marker *FOXG1*. The iCOs also expressed various neural markers (including *MAP2*, *NeuN*, and *TUJ1*), the deep-layer neuron marker *TBR1*, the neural progenitor cell marker *Nestin*, and the glutamate transport marker *vGLUT1* (Figure 3C). The protein expression of different neuronal and brain region-specific markers in the differentiated iCOs was confirmed. The co-localization of *SOX2* and *TUJ1* led to rosette formation within the organoid (Figure 4D). The co-localization of *TBR1* and *MAP2* mostly occurred near the edge of the iCOs, indicating the differentiation of the multiple layers of neurons (Figure 4E). *FOXG1* and *PROX1* represented the forebrain and hippocampal markers in the iCOs, respectively (Figure 4F). Collectively, these findings suggested that differentiated cerebral organoids recapitulate the protein expression patterns typically observed in different regions of the brain as well as in different types of neurons.

### Dose-Dependent Treatment With Estrogen Increased the Expression of Neuronal Markers in Induced Pluripotent Stem Cells-Derived Cerebral Organoids

To develop a model of the brain of patients with AD, we treated the iCOs with Aβ peptides and investigated whether estrogen protects neurons in Aβ peptide-treated iCOs (Aβ group) (Figure 4A; three iCOs used per experiment). The concentrations of estrogen used were determined from past research (Shum et al., 2015; Guo et al., 2018). The expression of the pluripotency marker *OCT*4 was significantly low in the iCOs in all groups (Figure 4B). The expression level of the neuronal marker *TUJ1* was significantly higher in iCOs treated with Aβ and 1 nM estrogen (EL group) than in those in the Aβ group (t(4) = 3.239, *p* = 0.02). The difference was more significant when the iCOs were treated with Aβ and 10 nM estrogen (EH group) (t(4) = 17.86, *P* < 0.001) (Figure 4C). Therefore, estrogen treatment enhanced the expression of neuronal markers. Conversely, *APP* expression increased significantly in both EL (t(4) = 3.325, *p* = 0.01) and EH (t(4) = 2.909, *p* = 0.02) groups compared to that in the Aβ group (Figure 3D). This indicates that estrogen may attenuate the effects of Aβ neurotoxicity. *ADAM10* expression was significantly high in the EL group (t(4) = 16.85, *P* < 0.001) and EH group (t(4) = 7.603, *P* < 0.001) (Figure 3E). These results indicated the potential protective effect of estrogen against neurotoxic Aβ peptides as well as its ability to enhance protein expression in neurons. To confirm that estrogen exerts protective effects on neurons in Aβ peptide-treated iCOs, we measured the expression of the early neuronal marker SOX2 and the neuronal marker TUJ1 (Figure 3F). The areas that stained for SOX2, TUJ1, and DAPI were measured. The expression of SOX2, TUJ1, and DAPI was significantly high in iCOs treated with Aβ and 1 nM estrogen, but it was higher in iCOs treated with 10 nM estrogen (Figures 4G,H). The results indicated that Aβ peptides are neurotoxic, and therefore, induce neuronal death in iCOs, and estrogen may alleviate the effects of Aβ peptides in a dose-dependent manner.

## Discussion

In this study, we evaluated the potential of cerebral organoids treated with specific concentrations of Aβ peptide as a model of AD. The organoids were treated to determine whether they could serve as an effective drug screening platform and yield results similar to those obtained in *in vivo* experiments. Estrogen reduced the degree of neurotoxicity in the iCOs, which indicated the potential of this model as an *in vitro* drug screening platform. Estrogen-treated AD mice performed better in the Barnes maze test than untreated AD mice (Figure 1). Furthermore, histological analyses of brain samples showed the significant suppression of AD-related pathological proteins, such as APP, Aβ peptide, and pTAU (Figure 2), in response to estrogen treatment. Given that estrogen attenuates the neurotoxic effects of Aβ peptides, we attempted to develop an *in vitro* model using cerebral organoids. After confirming the successful generation of iCOs differentiated from iPSCs (Figure 3), we treated the AD-recapitulating iCOs with estrogen and confirmed the enhancement of neuronal marker expression at both gene and protein levels (Figure 4).

While several studies have conducted *in vitro* and *in vivo* analyses on AD models and demonstrated the efficacies of therapeutic strategies in experimental models, the strategies have failed to yield effective results in clinical trials (Zahs and Ashe, 2010). Most cases of AD are sporadic (sporadic AD, SAD), although familial cases, which are usually associated with accelerated amyloidopathy, are considerably more aggressive, with earlier disease onset (Walsh and Selkoe, 2007). Hence, animal models of AD are genetically engineered to induce the overexpression of genes that are implicated most commonly in FAD, such as *APP* and *PS1* (encoding presenilin 1, which is the catalytic component of the Aβ peptide-producing complex gamma secretase) (Sasaguri et al., 2017; Gerakis and Hetz, 2019).

For sporadic AD is not only caused by genetic factors but also is caused by other risk factors, such as diabetes and hypertension, there is no established mouse models for sporadic AD (Foidl and Humpel, 2020). Hence, in the present study, we used a 5xFAD mouse model, which is a commonly used mouse model of AD, to study the effect of estrogen treatment on AD in menopausal patients (Ohno et al., 2007; Jawhar et al., 2012; Eimer and Vassar, 2013). Reportedly, the body weights of mice increase after ovariectomy, which is characteristic of the post-menopausal state (Blaustein et al., 1976; Gambacciani et al., 1997; Jeong and Yoon, 2012; Nishio et al., 2019). Similar results were reported in this study, since the body weights of ovariectomized mice were significantly higher than that of control mice (Figure 1B). Owing to the inhibition of estrogen release after ovariectomy, the thickness of the uterine wall is reduced; however, the thickness was shown to be restored partially with estrogen treatment (Kang et al., 2017). To confirm the successful delivery of estrogen, we stained the coronally dissected uterine horns of mice (Figure 1D). Compared to mice that were ovariectomized but untreated, mice treated with estrogen showed a significantly greater uterine wall thickness.

Although the transgenic mice model is invaluable for studying AD, it has certain limitations. The most important limitation is the inability of the model to mimic tau protein pathology, since only Aβ peptide generation cannot completely induce the formation of the central lesion observed in AD (Andorfer et al., 2003; Sasaguri et al., 2017). Additionally, while AD is a neurodegenerative disorder and occurs in aging brains, the histopathological characteristics of AD are observed early in transgenic mice, which significantly limits investigations on the pathogenesis of aging in these mice (Kitazawa et al., 2012). Therefore, an alternative disease modeling platform is necessary to study the pathological mechanisms underlying AD that involves central lesions.

To overcome the abovementioned limitations, we used iPSCs to develop cerebral organoids, which are self-organizing and self-developing three-dimensional structures that recapitulate certain characteristics of the brain (Lancaster et al., 2013). Cerebral organoids have been used to study neurodevelopmental disorders such as microcephaly (Lancaster et al., 2013) and autism spectrum disorder (Mariani et al., 2015; Birey et al., 2017). Cerebral organoids were first used as a model for late-onset AD recently. Before iCOs were used, neurons developed from iPSCs isolated from patients with FAD and SAD were used to demonstrate high Aβ peptide accumulation, tau phosphorylation, and endosomal alterations, all of which are early characteristics of AD (Cataldo et al., 2000; Israel et al., 2012; Muratore et al., 2014). Subsequently, human neuronal progenitor cells that were genetically engineered to overexpress mutant APP and PS1 were differentiated into cerebral organoids to develop a three-dimensional *in vitro* model of AD (Choi et al., 2014). This approach led to increased Aβ peptide accumulation in extracellular environments and tau phosphorylation with subsequent insoluble fibril formation. The field of utilizing cerebral organoids to study Alzheimer’s disease is expected to expand as the tissue engineering and bioengineering technologies advance (Ranjan et al., 2018). For example, microfluidics and spinning bioreactors are implemented in culturing cerebral organoid, which would allow more controlled neural patterning and support the delivery of oxygen and nutrient into the interior of the organoid (Kadoshima et al., 2013; Qian et al., 2016; Lancaster et al., 2017; Yan et al., 2019).

Despite technological advancements, there remain several limitations in the use of iCOs in investigations on AD. An important limitation is related to aging. Aging is a major risk factor in the pathogenesis of AD, as the process of aging is accompanied by different types of genetic alterations (Lopez-Otin et al., 2013; Gerakis and Hetz, 2019). However, as cerebral organoids derived from iPSCs mimic the prenatal brain, it is challenging to replicate the aging-related phenotypes of AD using cerebral organoids (Camp et al., 2015). Additionally, the cerebral organoid-based models currently in use do not show complete vascularization, owing to which the blood-brain barrier is not completely mimicked (Huch et al., 2017), and certain types of neuronal cells, such as oligodendrocytes, are not formed. Therefore, in this study, we attempted to study a single characteristic of AD—Aβ-related pathogenesis. We considered that if we treat healthy organoids with Aβ peptides, we could focus on the interplay between the neuronal cells of iCOs and Aβ peptides, which would mimic the interaction between neurons and Aβ plaques that occurs in the brains of patients with AD.

Another limitation is the low reproducibility and homogeneity of the model, because the generation of iCOs is heavily dependent on the self-organization potential of iPSCs (Papaspyropoulos et al., 2020). Accurate disease modeling is necessary to provide a stable platform; however, some cerebral organoids may produce higher concentrations of Aβ peptides than other organoids, leading to variability. We attempted to limit the variability in Aβ peptide production by manually treating the organoids with Aβ peptides. As shown in Figure 3, we successfully generated iCOs that recapitulated different parts of the brain as well as neuronal cell types.

In conclusion, we confirmed that estrogen exerts a protective effect against the toxicity induced by Aβ peptides and pTAU. We also supported this finding by modeling an *in vitro* platform that recapitulates Aβ plaque accumulation, one of the primary characteristics of AD. Previous studies have shown the toxic effects of Aβ peptides on neurons (Mamada et al., 2015; Tanokashira et al., 2017) and mouse brains (Kwakowsky et al., 2016), although no study has confirmed this in a three-dimensional platform based on stem cells. We treated iCOs obtained from healthy individuals with equal concentrations of Aβ peptide to mimic Aβ-induced neurotoxicity commonly observed in the brains of patients with AD (Figure 4). The organoids were then treated with estrogen in a dose-dependent manner to evaluate whether estrogen alleviated Aβ-induced neurotoxicity.

In our study, we focused on an estrogen receptor that is known to be affecting Alzheimer’s disease—α-secretase, specifically ADAM10. There are β- and γ-secretase, which are drug targets for AD, though it has been challenging to develop clinically appropriate drugs that target these proteases (Fahrenholz, 2007). Hence, we have decided to focus on α-secretase, for α-secretase has been a new therapeutic approach to targeting AD (Lichtenthaler, 2011). Current studies, as well as our own research, confirm that the treatment of estrogen may be therapeutically targeted as it promotes higher expression of alpha-secretase (Nord et al., 2010) and binds to estrogen receptors, though the exact mechanism is unclear to this day (Lan et al., 2015). There has been a study that focused on the regulatory effect of estrogen receptor alpha on ADAM9 (one of the candidates for alpha-secretase (Asai et al., 2003) that showed that activation of estrogen receptor alpha increased the expression of ADAM9 (Shen et al., 2016).

ADAM10 is known to be the main alpha-secretase that cleaves amyloid precursor protein (APP) into soluble forms of β-amyloid peptide, hence inhibiting the formation of β-amyloid peptide aggregation (Yuan et al., 2017; Peron et al., 2018; Sogorb-Esteve et al., 2018; Manzine et al., 2019). ADAM10, which is likely to activate alpha-secretase, may be regulated by protein kinase C (PKC) (Allinson et al., 2003). While estrogen binds to estrogen receptors, it also binds to PKCα and PKCδ (Alzamora et al., 2007). PKCα encourages alpha-secretase to undergo APP processing instead of BACE, yielding decreased amyloid-beta levels (Anastasio, 2013). Hence, higher expression of ADAM10 indicates lower probability of AD. In our study, we showed that treatment of estrogen induced higher expression of ADAM10 in both *in vivo* and *in vitro* settings (Figures 2B, 4D), as well as lowered level of amyloid-beta plaques and APP in *in vivo* setting (Figures 2B–D). Additionally, neuronal marker expression was enhanced at both the RNA and protein levels in response to estrogen treatment. Our findings suggest that iCOs can be used to study specific phenotypes of AD; the findings also suggest the potential of iCOs in the development of drug screening platforms. Future studies could involve the usage of different devices, such as atomic force microscopy, to accurately measure the sizes of the cerebral organoids in order to provide more accurate and stabilized experimental set-up.

## Data Availability Statement

The original contributions presented in the study are included in the article/Supplementary Material, further inquiries can be directed to the corresponding author/s.

## Ethics Statement

The animal study was reviewed and approved by Institutional Animal Care and Use Committee of the School of Medicine, The Catholic University of Korea.

## Author Contributions

JYK and JHJ contributed to conception and design of the study. JYK, HM, and JWK performed the experiments and organized the database. JYK and YN analyzed the data performed the statistical analysis. JYK wrote the first draft of the manuscript. JYK and YR wrote and edited the final manuscript. All authors contributed to manuscript revision, read, and approved the submitted version.

## Conflict of Interest

JK and YN are employed by YiPSCELL, Inc. JHJ is founder of YiPSCELL, Inc. The remaining authors declare that the research was conducted in the absence of any commercial or financial relationships that could be construed as a potential conflict of interest.

## Publisher’s Note

All claims expressed in this article are solely those of the authors and do not necessarily represent those of their affiliated organizations, or those of the publisher, the editors and the reviewers. Any product that may be evaluated in this article, or claim that may be made by its manufacturer, is not guaranteed or endorsed by the publisher.

## Footnotes

1
http://www.alz.org

## Supplementary Material

Supplementary Figure 1 Cerebral organoids harvested on day 60 showed greater expression of brain-relevant genes compared to those harvested on day 30. **(A)** Average gene expression normalized to that of *GAPDH*: pluripotency marker *OCT4*, hippocampal marker *PROX1*, forebrain marker *FOXG1*, neuronal markers *MAP2*, *NeuN*, and *TUJ1*, deep-layer neuron marker *TBR1*, neural progenitor cell marker *Nestin*, and glutamate transporter marker *vGLUT1*; gene expression was measured in cerebral organoids harvested on day 30 (M1) and day 60 (M2) (n = 3 for each group).

Supplementary Figure 2 Recording of Barnes maze test performed by mice.
