# Supplementary figures and images for: Mitigating Effect of Estrogen in Alzheimer’s Disease-Mimicking Cerebral Organoid
Source: Front Neurosci. 2022 Mar 24;16:816174. doi: 10.3389/fnins.2022.816174 (PMC8990972; doi:10.3389/fnins.2022.816174)

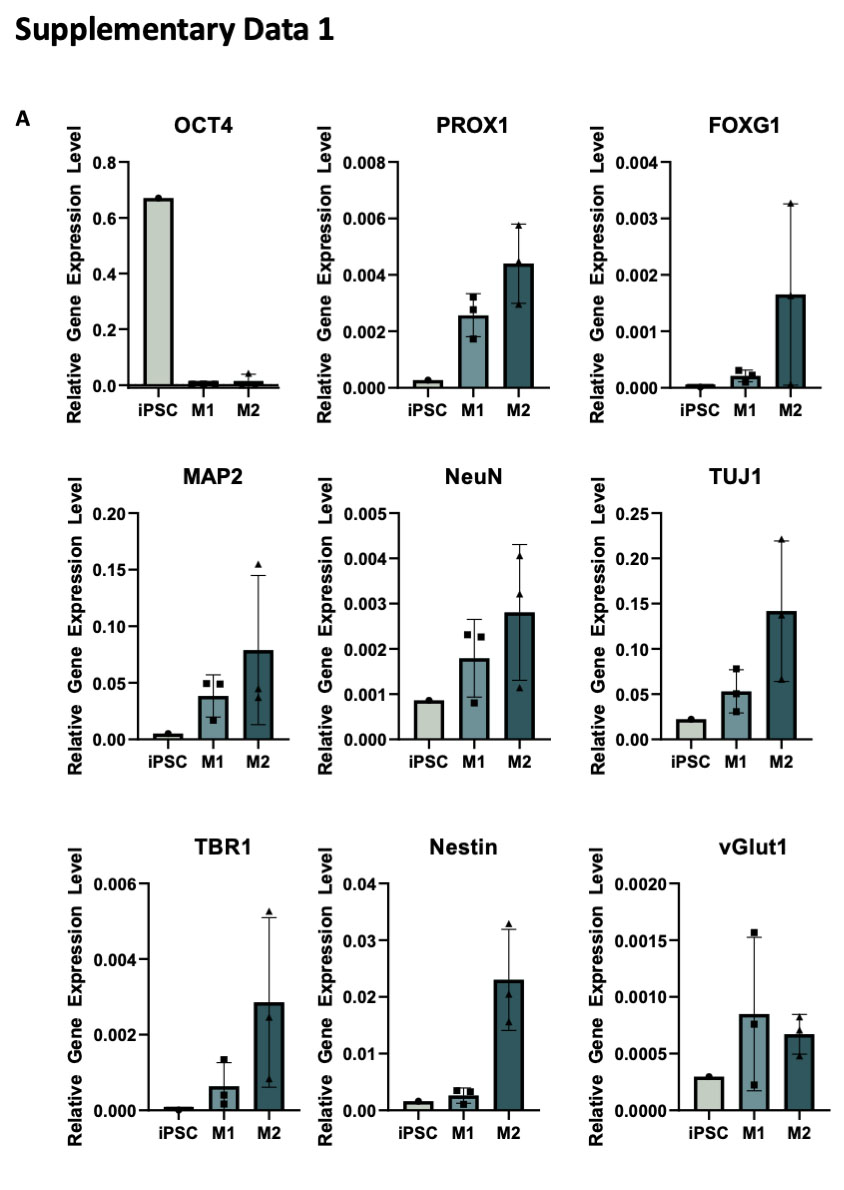

Supplement: Supplementary Figure 1 — Cerebral organoids harvested on day 60 showed greater expression of brain-relevant genes compared to those harvested on day 30. (A) Average gene expression normalized to that of GAPDH: pluripotency marker OCT4, hippocampal marker PROX1, forebrain marker FOXG1, neuronal markers MAP2, NeuN, and TUJ1, deep-layer neuron marker TBR1, neural progenitor cell marker Nestin, and glutamate transporter marker vGLUT1; gene expression was measured in cerebral organoids harvested on day 30 (M1) and day 60 (M2) (n = 3 for each group). [file Image_1.JPEG]

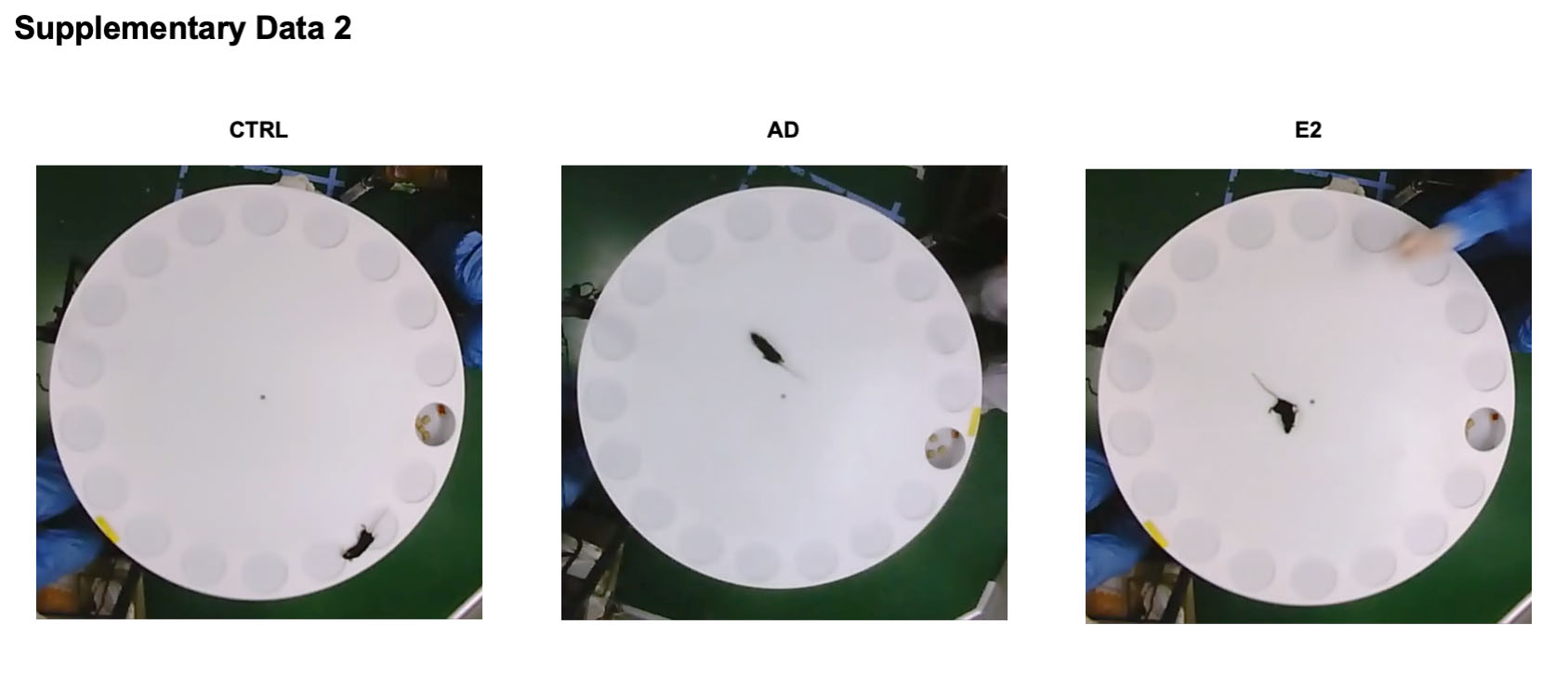

Supplement: Supplementary Figure 2 — Recording of Barnes maze test performed by mice. [file Image_2.JPEG]
